# Pesticides Burden in Neotropical Rivers: Costa Rica as a Case Study

**DOI:** 10.3390/molecules26237235

**Published:** 2021-11-29

**Authors:** Silvia Echeverría-Sáenz, Manuel Spínola-Parallada, Ana Cristina Soto

**Affiliations:** 1 Doctorado en Ciencia Naturales de para el Desarrollo (DOCINADE), Instituto Tecnológico de Costa Rica, Universidad Nacional, Universidad Estatal a Distancia, Heredia 40101, Costa Rica; 2 Central American Institute for Studies in Toxic Substances (IRET), Universidad Nacional, Heredia 86-3000, Costa Rica; 3 Instituto Internacional de Conservación y Manejo de Vida Silvestre, Universidad Nacional, Heredia 86-3000, Costa Rica; romeo.spinola.parallada@una.ac.cr; 4 Colaboratorio Nacional de Computación Avanzada (CNCA), Centro Nacional de Alta Tecnología (CeNAT), San José 10109, Costa Rica; csoto@cenat.ac.cr

**Keywords:** aquatic biodiversity, msPAF, lotic ecosystems, ERA, pesticides

## Abstract

Neotropical ecosystems are highly biodiverse; however, the excessive use of pesticides has polluted freshwaters, with deleterious effects on aquatic biota. This study aims to analyze concentrations of active ingredients (a.i) of pesticides and the risks posed to freshwater Neotropical ecosystems. We compiled information from 1036 superficial water samples taken in Costa Rica between 2009 and 2019. We calculated the detection frequency for 85 a.i. and compared the concentrations with international regulations. The most frequently detected pesticides were diuron, ametryn, pyrimethanil, flutolanil, diazinon, azoxystrobin, buprofezin, and epoxiconazole, with presence in >20% of the samples. We observed 32 pesticides with concentrations that exceeded international regulations, and the ecological risk to aquatic biota (assessed using the multi-substance potentially affected fraction model (msPAF)) revealed that 5% and 13% of the samples from Costa Rica pose a high or moderate acute risk, especially to primary producers and arthropods. Other Neotropical countries are experiencing the same trend with high loads of pesticides and consequent high risk to aquatic ecosystems. This information is highly valuable for authorities dealing with prospective and retrospective risk assessments for regulatory decisions in tropical countries. At the same time, this study highlights the need for systematic pesticide residue monitoring of fresh waters in the Neotropical region.

## 1. Introduction

Neotropical regions are recognized worldwide for their biodiversity. Antonelli and Sanmartín [1] stated this is “the most species rich region on Earth”, and Costa Rica is not the exception. According to data from the State of the Environment Report [2], the country has 5% of the world’s biodiversity. However, the same report and [3] consider that although the country has managed to make good decisions in conservation, one of the central oversights in terms of environmental protection has been the management of agrochemicals, their excessive use, and their contaminating effects on the different environmental compartments (air, water, soil), as well as on wildlife and human health [4,5].

As stated by FAO data [6], Costa Rica used 22.9 kg/a.i./ha in 2016 and uses more than 20 kg/a.i./ha/year since the year 2000. This figure represents the third-highest use in the world, much higher than the use of European countries (e.g., The Netherlands 10.02, Belgium 6.89, and Germany 3.92 kg/a.i./ha in 2016) and also much higher than other countries in the Neotropical region (Colombia 13.17, Ecuador 12.36, Guatemala 10.02, Belize 8, El Salvador 5.95, Brazil 4.31, and Nicaragua 2.47 kg/a.i./ha in 2016). This situation is reflected in freshwater contamination by pesticide residues. In Costa Rica, pesticide residues have been detected in various geographical regions of the country, including the Caribbean [4,5,6,7,8,9,10,11], the northern zone [12], the North Pacific [13,14,15], the South Pacific [16], and the horticultural areas of Pacayas and Zarcero in the Central Volcanic Mountain Range [17,18]. In the last 10 years, Cornejo et al. [19,20] also detected several pesticide residues in Panama, Barizon et al. [21] in Brazil, Hernández et al. [22] in Colombia, Deknock et al. [23] in Ecuador, Leyva Morales et al. [24] in Mexico, and Cárdenas et al. [25] in Venezuela.

Tropical climates have the advantage of allowing year-round cultivation, but this implies the year-round application of agrochemicals as well. Therefore, pesticides become “pseudo-persistent” and recurrent water pollutants [26] because, even though the half-life of many pesticides is short, and they could be degraded in a few days, the high application rates in the field, result in the detection of these substances in water bodies almost permanently. For example, [11] showed that the fungicide pyrimethanil and the herbicide diuron have a detection frequency of almost 90% in the water samples from the Madre de Dios River basin, while the insecticide ethoprophos and the fungicide epoxiconazole have frequencies of more than 70%. Very high detection frequencies (>50%) are also common in other areas of the country, with different active ingredients, varying according to the predominant crops [10,27].

It is clear that monocultures (especially genetically modified crops) have expanded greatly in Latin American countries, and with this increment, higher use of pesticides has also occurred [28]. In Central America, more than 180,000 tons of 353 a.i. were imported between the years 2005 and 2009 [29], and even though not all of the imported pesticides are used in the same area, it is clear that a considerable amount of toxic substances are being released into the environment regularly in Neotropical countries.

When these substances enter water bodies, they interact with the abiotic and biotic components of the ecosystem. The interaction with biota involves processes of entry, metabolization, and/or accumulation in organisms, which can produce direct or indirect deleterious effects [30,31,32]. In events of severe contamination, it is expected that species or entire groups of organisms that are more sensitive or lack escape mechanisms will disappear [33,34]. Therefore, the concentration or toxicity of pesticides themselves may explain much of the variation in aquatic species community structure even at regional scales [35,36].

Stehle and Schulz [37] present information that indicates that the richness of macroinvertebrate families was reduced ~30% in the presence of pesticide concentrations that represent acceptable limits at the regulatory level and that it is possible to observe a reduction of up to 63% in sites with concentrations that exceed acceptable limits. The same authors refer to information that reports concentrations of insecticides that exceed the regulatory limits. Therefore, it is noteworthy to indicate that this situation is widespread and that aquatic organisms are exposed to unacceptable concentrations of pesticides, mainly in tropical countries, where protection measures are laxer and the use of pesticides has increased.

For this reason, this study gathered the data from 11 research projects carried out in 5 different regions of Costa Rica, as a case study to generate information on the detection frequency, toxicity, and retrospective environmental risk of pesticides measured in field samples from more than 160 sites. We aimed to reflect the conditions of Neotropical agriculturally influenced rivers and calculate the potential effects of that pesticide burden on the biota of such aquatic ecosystems.

## 2. Results and Discussion

### 2.1. Pesticide Detection and Frequency

With the collection and digitalization of the information presented in Table S1, a unified database was generated. This database contains the results of pesticide residue analyses for 1036 water samples taken throughout Costa Rica.

The pesticide residue analysis database reveals 85 different active ingredients (a.i) or degradation products that were analyzed in the water samples. From these, 72 were detected (Table 1). Amongst the analyzed (but not detected a.i.) are bifenthrin and deltamethrin (pyrethroid insecticides), cyproconazole, and fenbuconazole (triazole fungicides), fenthion and malathion (organophosphates), as well as various metabolites of organochlorine pesticides such as PCP, PCNB, DDT, and endosulfan. The majority of these organochlorine pesticides have already been forbidden or restricted in Costa Rica since 1999 and 2005 (SFE, 2020); however, their degradation products are still detectable in other environmental matrices (dust, air, [38]). Pérez-Maldonado et al. [39] also assessed DDT levels in samples from México and Central America, detecting both DDT and DDE metabolites in soil, fish tissue, and children’s blood.

The 72 detected a.i are representatives of several biocide actions and chemical groups, including triazole, benzimidazole, aromatic hydrocarbon, pyridine, imidazole, and chlorinated fungicides; triazine, uracil, urea, oxazolidinone, and triazinone herbicides; organophosphate, organochlorine, pyrethroid, carbamate, thiadiazine, and neonicotinoid insecticides; as well as other acaricides, nematicides, among others They are also representative of a great diversity of toxic modes of action, which is presented in Table S2.

There are some herbicides—namely, diuron and ametryn; fungicides pyrimethanil, flutolanil, azoxystrobin, epoxiconazole, and myclobutanil; insecticides diazinon, buprofezin, chlorpyrifos, and ethoprophos for which high detection frequencies (≥20%) are observed at a national scale (Table 1). Furthermore, there are four forbidden substances (lindane, hexachlorobenzene, carbofuran, and bromacil) that were detected in water samples. Lindane and hexachlorobenzene were forbidden since 1999 and 2005, respectively; therefore, the detections imply illegal use of these pesticides in the mountain horticulture regions of the Central Volcanic Range. On the other hand, carbofuran, which was forbidden in 2014, was detected mostly prior to that year; however, one detection was registered in 2016. This could be the result of the use and application of product remnants already in existence (imported before the ban), but this would be highly improbable for the present and future years and should be analyzed with more detail by authorities since a high risk for aquatic biota has been demonstrated for this a.i. [7,18,27]. Bromacil is one of the most recently forbidden a.i. (2017), and it was also detected in posterior years (up to 2020); consequently, the risks associated with the potential lixiviation of this pesticide into groundwaters is still of concern, as it has been in other countries [40,41].

**Figure 1:**
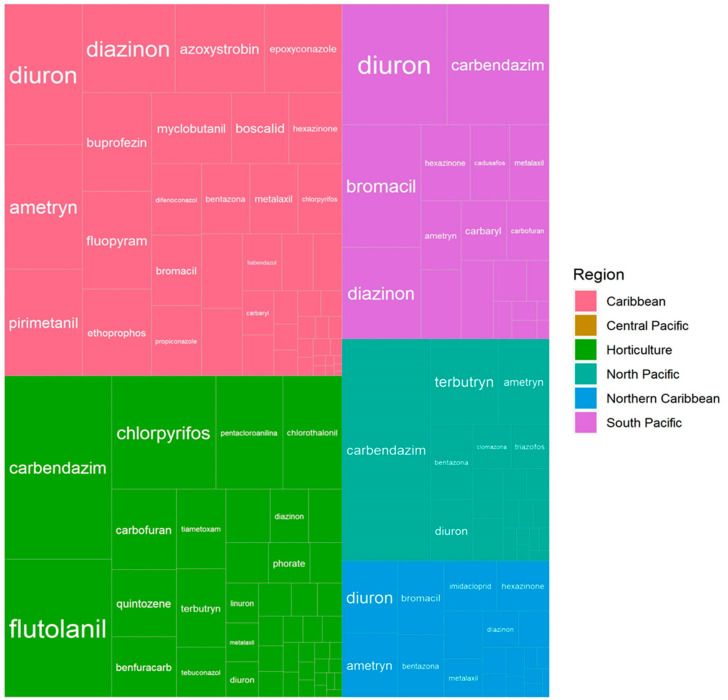
Detection frequency of pesticides in freshwater samples within different geographic regions of Costa Rica, between the years 2009 and 2019. Highest frequencies are located in the top left of each region box.

Differences in detection frequencies can be observed within regions in Costa Rica (Figure 1), with a higher frequency of fungicides in the Caribbean > mountain horticulture > South Pacific > North Pacific > Northern Caribbean > Central Pacific. Herbicides were more frequently detected in the South Pacific > Caribbean > North Pacific > Northern Caribbean > horticulture > Central Pacific, while insecticides and nematicides frequencies were highest in the mountain horticulture > Caribbean > South Pacific > Northern Caribbean > North Pacific > Central Pacific. It is noteworthy that the Central Pacific region has a considerably lower sampling effort than other areas, and almost no pesticides were detected in the analyzed samples; however, Rodríguez-Rodríguez et al. [42] conducted an intensive sampling (84 water samples) from 2008 to 2011 in melon and watermelon influenced catchments and found one fungicide and seven insecticides in concentrations that pose an acute and chronic risk to *Daphnia magna*, fish, and *Chironomus riparius*. This situation highlights the importance of increasing the sampling effort in that region. Furthermore, the highest individual pesticide frequencies were registered where more sampling effort has occurred; for example, for the horticulture mountain regions, chlorpyriphos was detected in 60% of the samples; in the South Pacific, diuron was detected in 64% and bromacil in 49% of the samples, while in the Caribbean, diuron, ametryn, pyrimethanil, diazinon, and azoxystrobin were detected in >40% of the samples.

**Figure 2:**
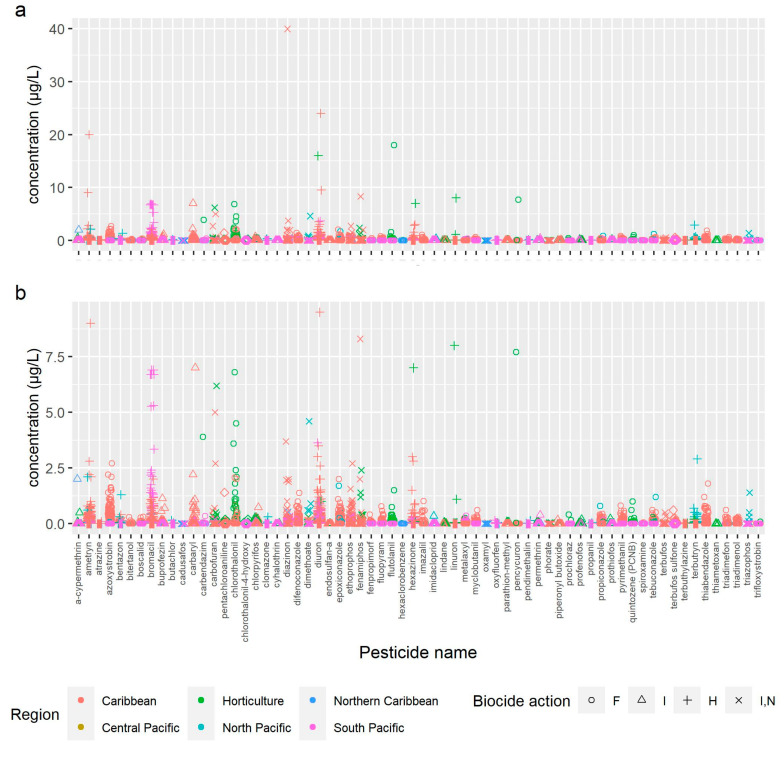
(**a**) Measured environmental concentration (MEC) of all pesticide active ingredients (a.i.) detected in freshwater Scheme 2009. (**b**) Zoom in of MEC <10 µg/L to increase clarity. F fungicide, I insecticide, H herbicide, I,N insecticide-nematicide.

Regarding the measured environmental concentration (MEC) of the a.i., Figure 2 shows all the field concentrations of 72 a.i. The majority of the pesticides were detected in concentrations <1 µg/L; however, in some cases, they reached values higher than 10 µg/L (e.g., diazinon, diuron, ametryn, and flutolanil), and at least 18 pesticides were >1 µg/L.

### 2.2. Comparison with International Regulations

We compared the mean and maximum detected concentrations with hazardous concentration 5% (HC_5_) and several international standards (EU-EQS, EPA water quality criteria, and the Australian and New Zealand Guidelines for Water Quality; Table 2). We also checked if the a.i are priority substances in the EU or US-EPA and if they were enlisted in the list of highly hazardous pesticides [43].

Available HC_5_ calculations reflect that those concentrations detected in field samples represent a risk for the biota of the aquatic ecosystems in Costa Rica. Likewise, 50% of the detected pesticides have mean and/or maximum concentrations that do not comply with one or more international standards (Table 2). Among the non-compliant a.i. are herbicides ametryn, bromacil, butachlor, diuron, hexazinone, oxyfluorfen, pendimethalin, and terbutryn; fungicides azoxystrobin, chlorothalonil, epoxiconazole, fenpropimorph, imazalil, pencycuron, and spiroxamine; insecticides cypermethrin, buprofezin, cadusafos, carbaryl, carbofuran, chlorpyriphos, cyhalothrin, diazinon, dimethoate, ethoprophos, fenamiphos, imidacloprid, lindane, phorate, profenofos, terbufos, and triazophos. Vryzas et al. [28] state that limitations in risk assessment, coupled with the low level of implementation of pesticide regulations are partially causing the presence of pesticides above the normative, which implies that environmental protection goals might not be reached.

It is valuable to mention that several of the non-compliant pesticides are also the ones with a higher frequency of detection (Table 1) and higher toxicity for aquatic organisms (e.g., the organophosphate and carbamate insecticides), and this should raise alarm about the conservation of aquatic ecosystems throughout the country. Additionally, we are aware that some highly used pesticides in Costa Rica (e.g., mancozeb, glyphosate, 2,4-D, among others) were not evaluated in this study because of analytical and methodological limitations, but for no reason must these results be interpreted as evidence that those a.i. do not exert effects on the aquatic ecosystems of the country.

### 2.3. Ecological Risk Multi-Substance Potentially Affected Fraction (msPAF) Model

Of the 85 pesticides detected in this study, 21 MoA were represented. These MoAs were further subdivided when species sensitivity distribution slopes (constructed with the toxicity data) of one a.i. differed more than 10% with respect to other a.i. that shared the same MoA (Table 3).

**Figure 3:**
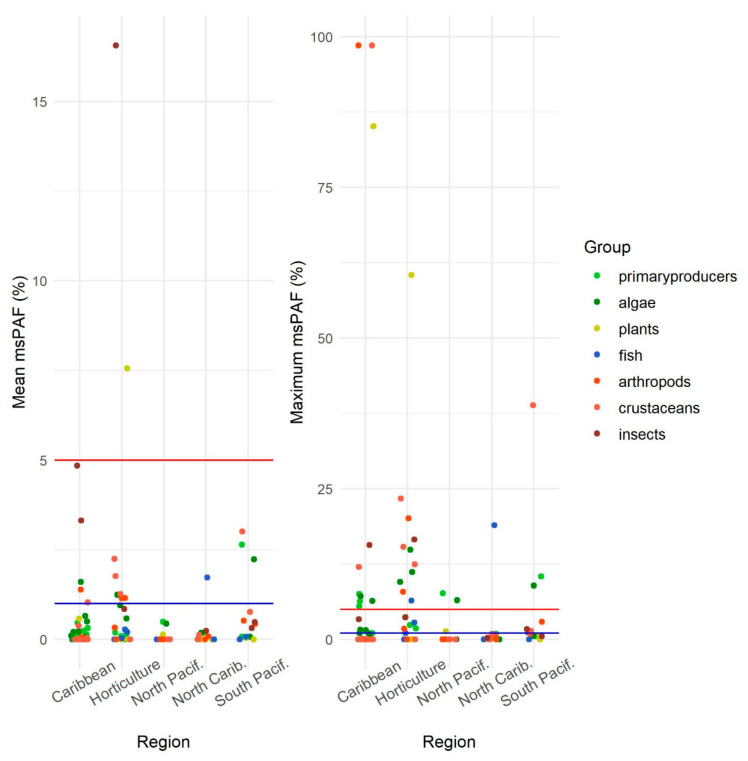
Mean and maximum multi-substance potentially affected fraction (msPAF) for 18 different watersheds within the studied regions in Costa Rica. Above the blue line (1% msPAF) risk is considered moderate; above red line (5% msPAF), risks are considered high.

We found that 5% and 13% of the total water samples from all regions of Costa Rica (except the Central Pacific, which had the least sampling effort) pose a high (msPAF > 5%) or moderate (msPAF > 1%) acute risk, respectively, especially to primary producers (plants, algae) and arthropods (insects, crustaceans). Figure 3 shows the mean and maximum msPAF, grouped by region. In the Caribbean, several samples had an extremely high risk for arthropods (insects and crustaceans) and aquatic plants, followed by the horticulture region, South Pacific, Northern Caribbean, and North Pacific.

**Figure 4:**
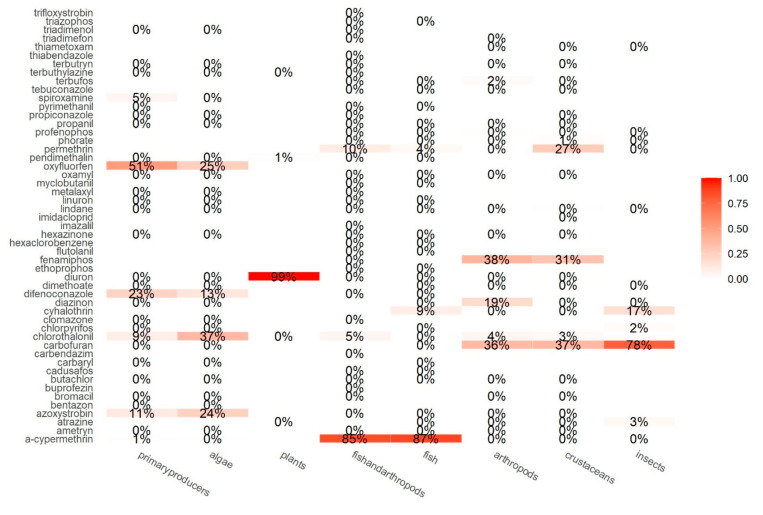
Fraction of the risk contributed by each pesticide in each species group.

The msPAF model illustrates the effect of the mixture of substances with different MoA in the analyzed water samples, but it is also possible to address the specific pesticides that contribute to the higher risks in each species group (Figure 4). Top risk contributors might pose a low risk on a frequent basis, or they might pose a high risk occasionally, or both.

In our study, herbicides diuron and oxyfluorfen, and fungicides azoxystrobin, chlorothalonil, difenoconazole, and spiroxamine are the top contributors to the risk posed on primary producers. Furthermore, diuron itself contributes to 99% of the cumulative risk on aquatic plants. The study by Rämö et al. [48] found the same result with diuron, suggesting that aquatic plants are more sensitive to this a.i. than algae, given that they have the same exposure data. It is noteworthy that the fungicides that are contributing to the risks on algae, fish, and arthropods have multisite action (chlorothalonil) or are ergosterol biosynthesis inhibitors, which is vital for all eukaryotic cells and, therefore, general enough to cause effects on non-fungi organisms [49]. All other imidazole or triazole fungicides have the same MoA [50] and could also potentially affect other groups of species. Regarding fish, a-cypermethrin, cyhalothrin, and permethrin (all pyrethroid insecticides), and fungicide chlorothalonil seem to be the a.i. posing the higher risks. Lastly, cyhalothrin and permethrin, as well as other organophosphate or carbamate insecticides (carbofuran, diazinon, fenamiphos, terbufos, chlorpyrifos) and fungicide chlorothalonil, are the higher contributors to the risk for arthropods (crustaceans, insects).

However, all these estimations are based on acute toxicity (EC50, LC50), and we cannot deny the fact that many other a.i. (such as organophosphates and carbamates) might be involved in chronic toxicity in all groups of species, but especially on fish, which require higher concentration exposures to show immobility or mortality endpoints but could be affected by the neurotoxic acetylcholinesterase inhibition properties of those insecticides [51,52].

Another relevant aspect is the presence of some high-risk pesticides identified in this study in other Neotropical countries. For example, ametryn in Ecuador [23]; azoxystrobin in Panama [19]; carbofuran in Brazil [21] and Panama; chlorpyrifos and diazinon in Ecuador, México [24], and Panama; diuron in Brazil, Colombia [22], and Ecuador; epoxiconazole in Colombia; ethoprophos in Panama; terbutryn in Ecuador. Furthermore, researchers in México and Venezuela [25] have detected very toxic pesticides such as aldrin, dieldrin, endrin, DDT, which are forbidden in many countries and are most likely posing unacceptable risks to the aquatic ecosystems.

We believe that greater efforts must be made by the government agencies and the farmers in the Neotropical region, in order to guarantee that toxic substances applied to the crops for pest control do not reach natural superficial waters in concentrations that pose unacceptable risks. The protection of the riparian vegetation is key to this purpose since it helps mitigate the effects of pesticides and excess nutrients to aquatic biota [53] and also provides habitat for refuge and later recolonization of organisms into the streams [54].

This study highlights the need for systematic pesticide residue monitoring of fresh waters in the Neotropical region, to acknowledge if the exposure to biota from specific pesticides is higher or lower than predicted by the risk analysis (toxicity tests and predictive models of exposure) executed prior to the registration [28]. Results from such a monitoring program would serve as a retrospective environmental risk assessment to address unacceptable risks.

## 3. Materials and Methods

### 3.1. Area of Study

Costa Rica is located between geographic coordinates 08°22′26″ and 11°13′12″ North latitude and 82°33′48″–85°57′57″ West longitude in the Central American Isthmus. Its climate is tropical, with a mean annual temperature of 26–27.6°C and mean annual precipitation of 1300 mm in the driest regions, up to a maximum of 7467 mm in the Grande de Orosi watershed [55]. Moreover, according to [56], Costa Rica harbors 12 different life zones (dry, moist, wet, and rain forests), distributed through several altitudinal ranges (lowland, premontane, lower montane, and montane), which lead to the high variability of temperature and rainfall throughout the country. In this study, we used superficial water samples retrieved from 160 sites throughout 5 different regions of Costa Rica (Caribbean, Northern Caribbean, North Pacific, Central Pacific, and South Pacific, as well as the mountainous horticultural zones of the Central Volcanic Range).

### 3.2. Database

We used previously generated information. The data (region, project, date, site, watershed, and pesticide residue analysis of 1036 water samples) were derived from 11 research projects carried out by state universities in the period between 2006 and 2019 (Table S1). All samples were analyzed in the Laboratory of Pesticide Residue Analysis at the National University (LAREP, IRET, UNA) or at the Center of Investigation on Environmental Pollution, at the University of Costa Rica (CICA, UCR). This assured uniformity of data quality irrespective of the year or the research project.

### 3.3. Pesticide Analysis

Surface water samples were collected by inserting pre-washed 2 L brown glass bottles into the water. The collected samples were transported in cooled ice boxes to the LAREP, IRET, UNA, or to the CICA, UCR, and stored at 4 °C for a maximum of 24 h before the analyses.

LAREP-UNA. Before 2018, pesticide analysis was performed as specified in Rämö et al. [40], while after that year, samples were analyzed by gas chromatography Agilent 7890A with mass detector 5975C (GC-MS) and liquid chromatography Waters Acquity UPLC H-Class with Waters XEVO T-QS Micro mass detector (UPLC-MS/MS). In both cases, a solid-phase extraction (SPE) was made prior to the analysis. For GC, the sample was agitated and passed through a previously conditioned Isolute ENV+ (200 mg/6 mL) cartridge, which was later dried and eluted with ethyl acetate. The extract was concentrated with Nitrogen and changed into Isooctane. Final volume of the extract was 0.25 mL. For UPLC, the same extraction procedure was followed, except that the elution was made with methanol, and it was concentrated into methanol/water (10:90 *v/v* or 40:60 *v/v*). The final volume of the extract was ~0.5 mL.

CICA. The method is a solid-phase extraction (SPE) and a liquid–liquid extraction (LLE) with dichloromethane, then solvent changes to acetone (for GC analysis), or with 0.1% formic acid in deionized water (for HPLC analysis). Afterward, a high-resolution multi-residue analysis in water samples by gas chromatography and liquid chromatography was used, as detailed in [13,18].

### 3.4. Comparison with International Regulations

We compared the mean and maximum detected concentrations of this study with environmental quality standards (EQS) from the European Union [44,47], the United States Environmental Protection Agency water quality criteria [45], the Deutsch Institute for Health and Environment maximum tolerable risk level [44], and the Australian and New Zealand Guidelines for Water Quality [46].

### 3.5. Ecological Risk Multi-Substance Potentially Affected Fraction (msPAF) Model

To complement the assessments derived by single-substance ecological risk, the msPAF model calculates the toxicity risk of mixtures of pesticides with known toxic modes of action (MoA). This model uses concentration addition (CA) to calculate a unique risk value for all the substances that have the same MoA and then applies response addition (RA) to summarize the toxicity risks of all different MoA. The outcome is a msPAF value that defines the potentially affected fraction (as a percentage) of a species group, resulting from the exposure to a complex mixture of pesticides [57,58].

For this study, to calculate the msPAF, we followed the methods described in detail by Rämö et al. [48]. However, we updated the information regarding the acute toxicity of each pesticide to aquatic biota, using new studies registered in the US Environmental Protection Agency (EPA) ECOTOX database [59]. Additionally, to assign MoA to each pesticide, we only used the classifications of the insecticide, fungicide, and herbicide resistance action committees [50,60,61]. We used the same 6 groups of organisms (algae, aquatic plants, arthropods, aquatic insects, crustaceans, and fish), we followed the same hazard unit calculation approach (geometric mean of toxicity data for each “species group-pesticide” combination), and we also set a minimum of 4 species’ toxicity data (in each species group–pesticide combination) to be included within the msPAF assessment. To interpret the results, a PAF < 1% is considered low risk, 1% > PAF < 5% is considered moderate, and PAF > 5% is interpreted as a high risk. Additionally, to address the specific pesticides that contribute to the higher risks in each species group, we followed the methods described by [48].

## 4. Conclusions

- Pesticides are ubiquitous contaminants of fresh waters in Costa Rica and other Neotropical countries;
- Several of the highly toxic active ingredients are detected in high frequencies (>20%) throughout Costa Rica, increasing the risks for aquatic biota;
- Concentrations at which individual analyzed pesticides are found in the country exceed criteria for biodiversity protection (HC_5_) and international standards, therefore representing a risk for the integrity and ecological functioning of aquatic ecosystems;
- msPAF reveals moderate and high risk derived from pesticide mixtures in water samples across Costa Rica;
- Pesticides consistently representing risk in Costa Rica (high frequency of detection, exceeding environmental standards, and identified as risk contributors within the msPAF model and literature) are a-cypermethrin, ametryn, azoxystrobin, bromacil. carbofuran, chlorothalonil, chlorpyrifos, diazinon, diuron, epoxiconazole, ethoprophos, fenamiphos, hexazinone, terbufos, and terbutryn;
- We believe these pesticides (except bromacil, which has already been forbidden) should be re-evaluated if their registration did not take into account current risk assessment tools;
- Several high-risk pesticides in Costa Rica are detected in other Neotropical countries;
- Deeper analysis of the responses of biota to the detected pesticides might be used to complement the development of numerical water-quality criteria and also for retrospective environmental risk evaluations for Neotropical countries;
- There is an urgent need for systematic pesticide residue monitoring of fresh waters in the Neotropical region.

## Figures and Tables

**Table 1:** Analyzed and detected pesticides from freshwater samples collected throughout Costa Rica between the years 2009 and 2019.

Active Ingredient	Num. of Analyzed Samples	Num. of Detections	Detection Frequency	Observations	Year of Prohibition/Restriction
diuron	917	339	36.97	A	
ametryn	991	315	31.79		
pyrimethanil	549	170	30.97	A	
flutolanil	432	130	30.09	A	
pentachloroaniline (M)	216	62	28.70		
diazinon	1000	279	27.90	A	
azoxystrobin	602	158	26.25	A	
buprofezin	431	99	22.97		
epoxiconazole	822	180	21.90	A	
chlorpyrifos	1029	204	19.83	R	2007
myclobutanil	456	90	19.74		
ethoprophos	914	180	19.69	R	2007
fluopyram	296	53	17.91		
bromacil	967	149	15.41	F	2017
chlorothalonil	914	136	14.88	A	
hexazinone	979	135	13.79		
bentazone	293	39	13.31		
difenoconazole	725	91	12.55	A	
metalaxyl	919	114	12.40		
propiconazole	846	99	11.70	A	
boscalid	291	32	11.00	A	
fenpropimorph	401	40	9.98	A	
thiabendazole	637	56	8.79	A	
carbendazim	126	11	8.73	A	
terbutryn	930	77	8.28		
tebuconazole	779	54	6.93	A	
carbofuran	846	58	6.86	F	2014
quintozene (PCNB)	783	41	5.24		
terbufos sulfone (M)	746	38	5.09		
fenamiphos	999	50	5.01		
imidacloprid	173	8	4.62	A	
carbaryl	837	36	4.30		
clorotalonil 4-hidroxi (M)	125	4	3.20		
profenophos	179	5	2.79		
hexachlorobenzene	545	15	2.75	F	2005
imazalil	449	12	2.67	A	
lindane	151	4	2.65	F	1999
triadimenol	827	20	2.42	A	
oxifluorfen	688	15	2.18	A	
dimethoate	750	16	2.13	A	
terbufos	992	18	1.81	R	2007
triadimefon	803	14	1.74		
linuron	787	13	1.65		
clomazone	290	4	1.38	A	
triazophos	531	7	1.32	A	
oxamyl	166	2	1.20		
phorate	917	11	1.20		
permethrin	685	7	1.02	A	
carbofuran phenol (M)	846	8	0.95		
bitertanol	768	7	0.91	A	
prothiofos	660	6	0.91		
tecnazene	146	1	0.68		
a-cypermethrin	794	5	0.63	A	
piperonyl butoxide	164	1	0.61		
cadusafos	346	2	0.58		
terbuthylazine	834	4	0.48		
butachlor	633	3	0.47	A	
spiroxamine	460	2	0.43		
prochloraz	550	2	0.36	A	
parathion-methyl	842	3	0.36	R	2007
pendimethalin	654	2	0.31	A	
tolclofos-methyl	657	2	0.30		
trifloxystrobin	393	1	0.25	A	
pencycuron	801	2	0.25		
atrazine	953	2	0.21		
propanil	543	1	0.18	A	
cyhalothrin	685	1	0.15	A	
endosulfan-a	1003	1	0.10	F *	2015
metribuzin	1	1	100		
dimetomorph	5	4	80		
benfuracarb	5	1	20		
thiametoxan	5	1	20	A	
endosulfan-b	992	0	0		
deltametryn	727	0	0	A	
malathion	670	0	0		
bifenthrin	626	0	0		
fenthion	620	0	0		
cyproconazole	582	0	0	A	
fenbuconazole	439	0	0	A	
endosulfan sulfate	418	0	0		
pentachlorobenzene (M)	154	0	0		
pentachloroanisol (M)	147	0	0		
DDE-pp (M)	142	0	0		
DDD-pp (M)	134	0	0		
pp-DDE (M)	42	0	0		

* Prohibition refers to endosulfan, not to the metabolites. F Forbidden; https://www.sfe.go.cr/DocsStatusRegistro/Listado_de_prohibidos.pdf (accessed on 9 February 2021). R Restricted https://www.sfe.go.cr/DocsStatusRegistro/Listado_de_Restringidos.pdf (accessed on 9 February 2021). A Aerial application allowed https://www.sfe.go.cr/DocsStatusRegistro/Lista_productos_aplicacion_aerea.pdf (accessed on 9 February 2021). M Metabolite or degradation product.

**Table 2:** The detected maximum and mean concentrations of analyzed pesticides, as compared with HC_5_ and international guidelines. Marked in **bold** are a.i. with mean or maximum concentration exceeding HC_5_ or international regulations.

Active Ingredient.	Biocide Action	Mean Detected Conc. (µg/L)	Max. Detected Conc. (µg/L)	HC_5_ (µg/L)	AA-EQS (EU) (µg/L)	MAC-EQS (EU) (µg/L)	MTR eco (µg/L)	EPA (Chronic) (µg/L)	EPA (Acute) (µg/L)	Aust (µg/L)	HHP (PAN)	Priority (EU)	Priority (EPA)
a-cypermethrin	insecticide	**0.06**	**2**	1.77	0.00008	0.0006					YES	YES	
ametryn	herbicide	**0.27**	**20**	0.23			0.01						
atrazine	herbicide	0.09	0.09	nc	0.6	2				13		YES	
azoxystrobin	fungicide	**0.39**	2.7	43.7	0.02	4.1							
bentazone	herbicide	0.16	1.3	828	73	450							
bitertanol	fungicide	0.10	0.29	nc			0.31						
boscalid	fungicide	0.07	0.3	nc			0.55						
bromacil	herbicide	**0.66**	**6.9**	3.8			0.0068						
buprofezin	insecticide	0.06	**1.13**	nc			0.56						
butachlor	herbicide	**0.001**	**0.04**	nc			0.00023				YES		
cadusafos	insecticide	**0.03**	**0.03**	nc	0.023	0.023					YES		
carbaryl	insecticide	**0.60**	**7**	1.02			0.23	2.1	2.1		YES		
carbendazim	fungicide	0.13	0.34	11.7	0.6	0.6					YES		
carbofuran	insecticide	0.41	**6.2**	0.4			0.91			1.2	YES		
chlorothalonil	fungicide	**0.28**	**6.8**	6.2	0.06						YES		
chlorpyrifos	insecticide	**0.06**	**0.73**	0.108	0.03	0.1		0.041	0.083	0.01	YES	YES	YES
clomazone	herbicide	0.19	0.3	nc			0.56						
cyhalothrin	insecticide	**0.03**	**0.025**	nc			0.0003				YES		
diazinon	insecticide	**0.28**	**40**	0.2	0.037			0.17	0.17	0.01	YES		YES
difenoconazole	fungicide	0.15	1.38	100.9	0.76	7.8							
dimethoate	insecticide	**0.08**	**0.9**	1.25	0.07	0.7				0.15	YES		YES
diuron	herbicide	**0.43**	**24**	2.6	0.2	1.8				0.2	YES	YES	YES
endosulfan-a	insecticide	**0.03**	**0.03**	nc	0.005	0.01		0.056	0.22	0.2	YES	YES *	YES
epoxiconazole	fungicide	**0.19**	**2**	nc	0.19	1.8					YES		
ethoprophos	insecticide	**0.15**	2.7	3.1			0.063				YES		
fenamiphos	insecticide	**0.29**	**8.3**	0.8	0.012	0.027					YES		
fenpropimorf	fungicide	0.06	**0.4**	nc			0.22						
fluopyram	fungicide	0.16	0.78	nc	2.7	32							
flutolanil	fungicide	0.24	18	nc			22						
hexachlorobenzene	fungicide	0.01	0.02	nc	-	0.05				0.1	YES	YES *	YES
hexazinone	herbicide	0.22	**7**	6.1			0.56						
imazalil	fungicide	0.38	**1.01**	nc			0.87				YES		
imidacloprid	insecticide	**0.35**	**0.35**	0.52	0.0083	0.2					YES		
lindane	insecticide	**0.04**	**0.08**	nc	0.02	0.04		-	0.95	0.2	YES		
linuron	herbicide	0.025	0.025	nc	0.17	0.29					YES		
metalaxyl	fungicide	0.08	0.36	5530			46						
myclobutanil	fungicide	0.09	0.6	nc			55						
oxamyl	insecticide	0.06	0.06	nc			1.8				YES		
oxyfluorfen	herbicide	0.05	**0.15**	0.5							YES		
parathion-methyl	insecticide	0.08	0.09	nc	11						YES		
pencycuron	fungicide	1.97	**3.9**	nc			2.7						
pendimethalin	herbicide	**0.14**	**0.14**	3.26	0.018	0.024					YES		
permethrin	insecticide	**0.18**	**0.4**	nc			0.0003				YES		
phorate	insecticide	**0.03**	**0.05**	nc			0.00017				YES		YES
piperonyl butoxide	insecticide	0.17	0.17	nc									
prochloraz	fungicide	0.28	0.4	nc			1.3						
profenofos	insecticide	**0.13**	**0.2**	nc			0.00003			0.02	YES		
propanil	herbicide	0.025	0.025	12			0.07						
propiconazole	fungicide	0.10	1	386.8			10				YES		
prothiofos	insecticide	0.06	0.22	nc							YES		
pyrimethanil	fungicide	0.10	0.81	1740	7	33							
quintozene (PCNB)	fungicide	0.09	1	nc			3.1						
spiroxamine	fungicide	**0.05**	**0.05**	nc			0.002						
tebuconazole	fungicide	0.10	1.2	848.1	0.63	14					YES		
terbufos	insecticide	**0.03**	**0.5**	0.1			0.00003				YES		
terbuthylazine	herbicide	0.03	0.04	5.74	0.2	1.3							
terbutryn	herbicide	**0.10**	**2.9**	5.4	0.065	0.34						YES	
thiabendazole	fungicide	0.28	1.2	nc			3.3				YES		
thiametoxan	insecticide	0.025	0.025	nc	0.14						YES		
triadimefon	fungicide	0.28	0.6	754.3			0.91						
triadimenol	fungicide	0.17	0.31	2160			3.2				YES		
triazophos	insecticide	**0.03**	**0.5**	nc	0.001	0.02					YES		
trifloxystrobin	fungicide	0.08	0.08	nc	0.27	0.81							

* HC_5_: Hazardous concentration 5%; concentration of pesticide “x” that causes a toxic effect on 5% of the species, within a species sensitivity distribution (SSD). nc = not calculated [7]; Arias-Andrés pers. com. (2021). AA-EQS Annual average environmental quality standard for long-term exposure (chronic) [44]. MAC-EQS Maximum acceptable concentration environmental quality standard for short-term exposure (acute) [44]. MTR (Maximum tolerable risk) is the concentration of a substance in the environment below which no negative effect is expected. The MTR applies to long-term (chronic) exposure [44]. EPA (Chronic and acute) water quality criteria for aquatic life [45]. Australian and New Zealand guidelines for fresh and marine water quality (protection for 95% of the species; chronic) [46]. HHP (PAN) Highly hazardous pesticides according to the criteria from the “Pesticide Action Network” [43]. Priority (EU and EPA) refers to the priority substances enlisted by the European Union [47] and the Environmental Protection Agency of the United States of America [45].

**Table 3:** MoA assigned to each pesticide for the msPAF calculations. The subdivision of MoA is depicted with letters (a–d). Pesticides without an assigned TMoA for a species group were not assessed for that group in msPAF. Pesticides absent from this table did not have enough toxicity data to be incorporated in the model.

Active Ingredient	Biocide Action	MoA *	Algae	Aquatic Plants	Primary Producers	Insects	Crustaceans	Arthropods	Fish	Fish and Arthropods
metalaxyl	fungicide	FA1	1		1					1
carbendazim	fungicide	FB1								2
thiabendazole	fungicide	FB1								2a
flutolanil	fungicide	FC2							3	3
azoxystrobin	fungicide	FC3	4		4		4	4	4	4
trifloxystrobin	fungicide	FC3								4
pyrimethanil	fungicide	FD1			5				5	5
quintozene (PCNB)	fungicide	FF3								6
difenoconazole	fungicide	FG1	7		7b				7	7
imazalil	fungicide	FG1								7a
myclobutanil	fungicide	FG1							7a	7b
propiconazole	fungicide	FG1	7a		7a		7b			7a
tebuconazole	fungicide	FG1					7a	7a	7b	7a
triadimefon	fungicide	FG1						7b		7b
triadimenol	fungicide	FG1	7b		7a					7c
spiroxamine	fungicide	FG2	8		8					
chlorothalonil	fungicide	FM	9	9	9		9	9	9	9
clomazone	herbicide	H13	10		10					10
oxyfluorfen	herbicide	H14	11		11					
butachlor	herbicide	H15	12		12		12	12	12	12
pendimethalin	herbicide	H3	13	13	13				13	13
ametryn	herbicide	H5	14		14		14a	14a	14	14b
atrazine	herbicide	H5		14a		14	14a	14c	14	
bromacil	herbicide	H5	14		14a		14	14		14
diuron	herbicide	H5	14a	14b	14		14a	14a	14a	14b
hexazinone	herbicide	H5	14		14		14	14	14b	14a
linuron	herbicide	H5	14		14b				14	14a
propanil	herbicide	H5	14a		14		14a	14a	14	14
terbuthylazine	herbicide	H5	14	14	14a					14d
terbutryn	herbicide	H5	14a		14		14b	14b		14c
bentazon	herbicide	H6	15		15					
buprofezin	insecticide	I16								16
carbaryl	insecticide	I1A	17		17				17a	
carbofuran	insecticide	I1A	17a		17	17	17a	17a	17	
oxamyl	insecticide	I1A	17		17a		17	17	17	17
cadusafos	insecticide	I1B							18	18
chlorpyrifos	insecticide	I1B	18b		18	18a			18b	
diazinon	insecticide	I1B	18		18b	18	18a	18a	18	
dimethoate	insecticide	I1B	18a		18a	18a	18b	18b	18b	
ethoprophos	insecticide	I1B							18	18
fenamiphos	insecticide	I1B					18	18		18
parathion-methyl	insecticide	I1B	18b		18	18			18a	
phorate	insecticide	I1B				18	18b	18b	18b	18
profenophos	insecticide	I1B				18a	18a	18b	18	18
terbufos	insecticide	I1B					18a	18a	18	18
triazophos	insecticide	I1B							18b	18
endosulfan-a	insecticide	I2A					19	19	19	19a
lindane	insecticide	I2A	19		19	19	19a	19	19a	19
a-cypermethrin	insecticide	I3A	20		20	20	20	20	20a	20
cyhalothrin	insecticide	I3A				20a	20	20	20b	
permethrin	insecticide	I3A				20	20a	20	20	20a
imidacloprid	insecticide	I4A					21			
thiametoxam	insecticide	I4A				21	21	21		

* Corresponds to codification in [48,49,50] and the initial of the biocide action: F= fungicide; H = herbicide; I = insecticide.

## Data Availability

The data presented in this study are available on request from the corresponding author. The data are not publicly available yet, due to pending publication in a public repository.

## Supplementary Materials

Table S1: Data and information sources for the analysis, Table S2: Characteristics (CAS identification number, biocide action, chemical group, and mode of action) of the detected pesticides, as well as references to studies in which they have been stated as high-risk pesticides for the aquatic environment in Costa Rica. References [62,63] are cited in the Supplementary Materials.

## Sample Availability

Samples of the compounds are not available from the authors.
