# Supplementary material for: Pesticides Burden in Neotropical Rivers: Costa Rica as a Case Study
Source: Molecules. 2021 Nov 29;26(23):7235. doi: 10.3390/molecules26237235 (PMC8658955; doi:10.3390/molecules26237235)
Supplement: Supplementary file 1 [file molecules-26-07235-s001.zip › molecules-1453701-supplementary.pdf]

Supplementary information: Table S1. Data and information sources for the analysis.

Year	Project name (in spanish)	Reference (TR =Technical report; PA = Published article)
2009	Diagnóstico sobre contaminación de aguas, suelos y productos hortícolas por el uso de agroquímicos en la microcuenca de las quebradas Plantón y Pacayas en Cartago, Costa Rica.	[17] (TR)
2009-2010	Métodos biológicos para evaluar el estado ecológico de las comunidades ribereñas, en las zonas piñeras del Caribe de Costa Rica.	[10] (PA)
2009-2011	Impacto de los plaguicidas en el recurso hídrico de la cuenca del río Tempisque (Palo Verde) Costa Rica. Bases científicas para la gestión ambiental sostenible.	[62] (TR)
2011-2012	Identificación de amenazas y capacitación para el uso sostenible del Refugio Nacional de Vida Silvestre Caño Negro, Región Huetar Norte.	[12] (PA)
2011-2013	Plan de gestión de la cuenca del Río Volcán, Pacífico Sur de Costa Rica.	[16] (TR)
2011-2013	Evaluación del riesgo ecológico de la escorrentía de plaguicidas usados en la agricultura hacia el Río y la Laguna Madre de Dios en la zona del Caribe, Costa Rica "TROPICA".	[11] (PA)
2013-2015	Las buenas prácticas agrícolas en el uso y manejo de agroquímicos en la zona hortícola de Zarcero, Alajuela.	[27] (TR)
2016-2020	Plan Nacional de Monitoreo de la calidad de los cuerpos de agua superficiales (todo el país).	[63] (TR)
2011-2019	Observatorio Ambiental de la Universidad Nacional; Indicador: Presencia de residuos de plaguicidas y calidad biológica del Río Jiménez, Caribe de Costa Rica.	[63] (TR)
2018-2020	Efectos de la presencia de residuos de plaguicidas y otros factores ambientales, en el establecimiento de la comunidad de macroinvertebrados acuáticos en una quebrada con influencia piñera en el pacífico sur de Costa Rica.	[16] (TR)
2018-2020	Procesos de Gestión Integrada del Recurso Hídrico en las subcuencas Chiz-Maravilla y Quebrada Honda, Cartago, Costa Rica.	[63] (TR)

Supplementary information: Table S2. Characteristics (cas identification number, biocide action, chemical group and mode of action) of the detected pesticides, as well as references to studies in which they have been stated as high risk pesticides for the aquatic environment in Costa Rica.

Active ingredient	cas	High ecological risk	Biocide action	Chemical group	Entrance	FRAC/ HRAC /IRAC MoA	MoA Description
a-cypermethrin	52315-07-8	[12]; [27]; [18]; [42]	insecticide	pyrethroid	contact	I3A	Sodium channel modulator (blocks nervous stimuli)
ametryn	834-12-8	[7]; [48]; [11]	herbicide	triazine	systemic	H5	photosystem II inhibitor (D1 Serine 264 Binders)
atrazine	1912-24-9		herbicide	triazine	systemic	H5	photosystem II inhibitor (D1 Serine 264 Binders)
azoxystrobin	131860-33-8	[11]	fungicide	mathoxy-acrylate	systemic, translaminar	FC3	prevents mithocondrial respiration (cytochrome bc1 (ubiquinol oxidase) at Qo site (cyt b gene)
benfuracarb*	82560-54-1		insecticide	carbamate	systemic, contact	I1B	Acetylcholinesterase inhibitor
bentazon	25057-89-0		herbicide	benzothiadiazole	contact	H6	photosystem II inhibitor (D1 Histidine 215 Binders)
bitertanol	55179-31-2		fungicide	triazole	systemic	FG1	demethylation in sterol biosynthesis inhibitor
boscalid	188425-85-6		fungicide	pyridine-carboxamide	foliar	FC2	Succinate dehydrogenase inhibitor
bromacil	314-40-9	[7]; [48]; [11]	herbicide	uracil, bromated	systemic	H5	photosystem II inhibitor (D1 Serine 264 Binders)
buprofezin	69327-76-0		insecticide	thiadiazine	contact	I16	chitin biosynthesis type 1 inhibitor
butachlor	23184-66-9		herbicide	chloroacetamide	systemic	H15	Very long chain fatty acids inhibitor

Active ingredient	cas	High ecological risk	Biocide action	Chemical group	Entrance	FRAC/ HRAC /IRAC MoA	MoA Description
cadusafos	95465-99-9		insecticide, nematicide	organophosphate	contact	I1B	Acetylcholinesterase inhibitor
carbaryl	63-25-2	[48]; [11]	insecticide	carbamate	contact	I1A	Acetylcholinesterase inhibitor
carbendazim	10605-21-7	[13]; [18]	fungicide	benzimidazole	systemic	FB1	ergosterol synthesis inhibitor
carbofuran	1563-66-2	[7]; [27]; [18]; [42]	insecticide, nematicide	carbamate	systemic, contact	I1A	Acetylcholinesterase inhibitor
carbofuran phenol (carbofuran M)	1563-38-8		unclassified	unclassified	not applicable		not applicable
chlorothalonil	1897-45-6	[11]; [27]	fungicide	chloronitrile phthalonitrile	foliar, contact	FM	multi-site activity
chlorothalonil-4-hidroxy (M)	28343-61-5		unclassified	unclassified	not applicable		not applicable
chlorpyrifos	2921-88-2	[11]; [27]; [18]	insecticide	organophosphate	contact, respiratory	I1B	Acetylcholinesterase inhibitor
clomazone	81777-89-1		herbicide	oxasolidinone, chlorinated	systemic	H13	DXP synthesis inhibitor
cyhalothrin	91465-08-6		insecticide	pyrethroid, chlorinated, flourated	contact		neurotoxic
diazinon	333-41-5	[7]; [48]; [11]; [27]	insecticide, nematicide	organophosphate	contact, respiratory	I1B	Acetylcholinesterase inhibitor
difenoconazole	119446-68-3		fungicide	triazole	systemic	FG1	demethylation in sterol biosynthesis inhibitor
dimethoate	60-51-5	[27]; [13]	insecticide, nematicide	organophosphate	systemic, contact	I1B	Acetylcholinesterase inhibitor

Active ingredient	cas	High ecological risk	Biocide action	Chemical group	Entrance	FRAC/ HRAC /IRAC MoA	MoA Description
diuron	330-54-1	[12]; [7]; [48] [11]; [13]; [18]	herbicide	urea, chlorinated	systemic	H5	photosystem II inhibitor (D1 Serine 264 Binders)
endosulfan-a	959-98-8	[13]; [42]	insecticide	organochlorine	contact		GABA-gated chloride channel blockersneurotoxic
epoxiconazole	133855-98-8	[13]	fungicide	triazole	preventive	FG1	demethylation in sterol biosynthesis inhibitor
ethoprophos	13194-48-4	[12]; [7]; [48]; [11]	insecticide, nematicide	organophosphate	contact	I1B	Acetylcholinesterase inhibitor
fenamiphos	22224-92-6	[7]; [11]	insecticide, nematicide	organophosphate	systemic, contact	I1B	Acetylcholinesterase inhibitor
fenpropimorf	67564-91-4		fungicide	morpholine	systemic	FG2	sterol biosynthesis inhibitor
fluopyram	658066-35-4		fungicide	pyridinyl-ethyl-benzamide	preventive, systemic	FC2	Succinate dehydrogenase inhibitor
flutolanil	66332-96-5		fungicide	phenyl-benzamide	systemic	FC2	Succinate dehydrogenase inhibitor
hexaclorobenzene	118-74-1		fungicide	organochlorine	contact, ingestion, respiratory		unknown for fungi
hexazinone	51235-04-2	[11]	herbicide	triazinone	systemic, contact	H5	photosystem II inhibitor (D1 Serine 264 Binders)
imazalil	35554-44-0		fungicide	imidazole	systemic	FG1	demethylation in sterol biosynthesis inhibitor
imidacloprid	138261-41-3		insecticide	neonicotinoid, chlorinated	systemic, contact	I4A	nicotinic acetylcholine receptor competitive modulator

Active ingredient	cas	High ecological risk	Biocide action	Chemical group	Entrance	FRAC/ HRAC /IRAC MoA	MoA Description
lindane	58-89-9	[27]	insecticide	organochlorine	contact, respiratory	I2A	GABA-gated chloride channel blockersneurotoxic
linuron	330-55-2		herbicide	urea, chlorinated	systemic	H5	photosystem II inhibitor (D1 Serine 264 Binders)
metalaxyl	57837-19-1		fungicide	acylalanine	systemic	FA1	protein synthesis inhibitor
myclobutanil	88671-89-0		fungicide	triazole	systemic	FG1	demethylation in sterol biosynthesis inhibitor
oxamyl	23135-22-0	[18]	insecticide, nematicide	carbamate	systemic, contact	I1A	Acetylcholinesterase inhibitor
oxyfluorfen	42874-03-3		herbicide	diphenilether, chlorinated, fluorated		H14	Protoporphyrinogen Oxidase inhibitor
parathion-methyl	298-00-0		insecticide	organophosphate	contact	I1B	Acetylcholinesterase inhibitor
pencycuron	66063-05-6		fungicide	phenylurea	protective	FB4	celular division inhibitor
pendimethalin	40487-42-1		herbicide	dinitroaniline	systemic	H3	celular division(microtubule assembly) inhibitor during germination
pentachloroaniline (quintozene M)	527-20-8		unclassified	unclassified	not applicable		not applicable
permethrin	52645-53-1	[11]; [27]	insecticide	pyrethroid, chlorinated	contact	I3A	Sodium channel modulator (blocks nervous stimuli)
phorate	298-02-2	[27]	insecticide, nematicide	organophosphate	systemic, contact	I1B	Acetylcholinesterase inhibitor

**Active ingredient**	**cas**	**High ecological risk**	**Biocide action**	**Chemical group**	**Entrance**	**FRAC/ HRAC /IRAC MoA**	**MoA Description**
piperonyl butoxide	51-03-6		insecticide		increases efficacy of other insecticides (mostly pyrethroids)		oxidase inhibitor
prochloraz	67747-09-5		fungicide	imidazole	contact	FG1	demethylation in sterol biosynthesis inhibitor
profenophos	41198-08-7	[27]	insecticide	organophosphate, chlorinated, bromated	contact	I1B	Acetylcholinesterase inhibitor and ovicidal properties
propanil	709-98-8	[13]	herbicide	amide	contact	H5	photosystem II inhibitor (D1 Serine 264 Binders)
propiconazole	60207-90-1	[11]	fungicide	triazole	systemic	FG1	demethylation in sterol biosynthesis inhibitor
prothiofos	34643-46-4	[27]	insecticide	organophosphate, chlorinated	contact	I1B	Acetylcholinesterase inhibitor
pyrimethanil	53112-28-0		fungicide	anilino-pyrimidine	protective	FD1	methionine biosynthesis inhibitor
quintozene (PCNB)	82-68-8	[27]	fungicide	aromatic hydrocarbon	contact	FF3	possible lipid peroxidation
spiroxamine	118134-30-8		fungicide	spiroketal-amine	protective and systemic	FG2	sterol biosynthesis inhibitor
tebuconazole	107534-96-3		fungicide	triazole	systemic, contact	FG1	demethylation in sterol biosynthesis inhibitor
tecnazene	117-18-0		fungicide	aromatic hydrocarbon	protective and curative	FF3	possible lipid peroxidation

Active ingredient	cas	High ecological risk	Biocide action	Chemical group	Entrance	FRAC/ HRAC /IRAC MoA	MoA Description
terbufos	13071-79-9	[7]; [48]; [11]	insecticide, nematicide	organophosphate	contact	I1B	Acetylcholinesterase inhibitor
terbufos sulfone (terbufos M)	56070-16-7		unclassified	unclassified	not applicable		not applicable
terbuthylazine	5915-41-3		herbicide	triazine, chlorinated	systemic	H5	photosystem II inhibitor (D1 Serine 264 Binders)
terbutryn	886-50-0	[13]	herbicide	triazine	systemic	H5	photosystem II inhibitor (D1 Serine 264 Binders)
thiabendazole	148-79-8		fungicide	benzimidazole	systemic	FB1	mithosis inhibitor (β-tubulin assembly)
thiametoxam	153719-23-4		insecticide, nematicide	nicotinic, chlorinated	systemic, contact	I4A	nicotinic acetylcholine receptor competitive modulator
tolclofos-methyl	57018-04-9		fungicide	aromatic hydrocarbon	contact	FF3	possible lipid peroxidation
triadimefon	43121-43-3		fungicide	triazole	systemic	FG1	demethylation in sterol biosynthesis inhibitor
triadimenol	55219-65-3		fungicide	triazole	systemic	FG1	demethylation in sterol biosynthesis inhibitor
triazophos	24017-47-8	[13]	insecticide, nematicide	organophosphate	contact	I1B	Acetylcholinesterase inhibitor
trifloxystrobin	141517-21-7		fungicide	oximino-acetate	preventive	FC3	prevents mithocondrial respiration (cytochrome bc1 (ubiquinol oxidase) at Qo site (cyt b gene)

M = metabolite; Source for modes of action: [50, 60, 61]
